# Targeting *Staphylococcus aureus* Toxins: A Potential form of Anti-Virulence Therapy

**DOI:** 10.3390/toxins8030072

**Published:** 2016-03-15

**Authors:** Cin Kong, Hui-min Neoh, Sheila Nathan

**Affiliations:** 1 School of Biosciences and Biotechnology, Faculty of Science and Technology, Universiti Kebangsaan Malaysia, 43600 Bangi, Selangor Darul Ehsan, Malaysia; kong_cin@hotmail.com; 2 UKM Medical Molecular Biology Institute (UMBI), Universiti Kebangsaan Malaysia, 56000 Cheras, Kuala Lumpur, Malaysia; hui-min@ppukm.ukm.edu.my

**Keywords:** *Staphylococcus aureus*, toxins, virulence factors, regulatory system, anti-virulence therapy

## Abstract

*Staphylococcus aureus* is an opportunistic pathogen and the leading cause of a wide range of severe clinical infections. The range of diseases reflects the diversity of virulence factors produced by this pathogen. To establish an infection in the host, *S. aureus* expresses an inclusive set of virulence factors such as toxins, enzymes, adhesins, and other surface proteins that allow the pathogen to survive under extreme conditions and are essential for the bacteria’s ability to spread through tissues. Expression and secretion of this array of toxins and enzymes are tightly controlled by a number of regulatory systems. *S. aureus* is also notorious for its ability to resist the arsenal of currently available antibiotics and dissemination of various multidrug-resistant *S. aureus* clones limits therapeutic options for a *S. aureus* infection. Recently, the development of anti-virulence therapeutics that neutralize *S. aureus* toxins or block the pathways that regulate toxin production has shown potential in thwarting the bacteria’s acquisition of antibiotic resistance. In this review, we provide insights into the regulation of *S. aureus* toxin production and potential anti-virulence strategies that target *S. aureus* toxins.

## 1. Introduction

The Gram-positive bacterium *Staphylococcus aureus* is a medically-important pathogen of a variety of human infections, ranging from relatively trivial superficial skin infections to deep-seated tissue infection and bacteremia. *S. aureus*, especially the antibiotic-resistant strains, is predominantly associated with high morbidity and mortality in nosocomial infections resulting from severe sepsis and septic shock. Methicillin-resistant *S. aureus* (MRSA) infections impose a significant burden on healthcare around the world, with higher mortality, morbidity, and financial costs compared to methicillin-susceptible *S. aureus* (MSSA). This pathogen has also been classified as a threat to both hospital and community settings, necessitating extra monitoring and prevention activities by the Centers for Disease Control and Prevention (CDC) [1]. *S. aureus* is notorious for its ability to acquire resistance to the commonly used antimicrobial agents as typified by MRSA, vancomycin-intermediate *S. aureus* (VISA), and vancomycin-resistant *S. aureus* (VRSA). The inexorable onslaught by antibiotic-resistant *S. aureus* that continues to threaten the community presents an urgent need for novel therapeutic approaches that do not exert selective pressure on evolutionary adaptation of the bacteria. An alternative approach is to develop anti-virulence therapies that interfere with bacterial toxins or virulence factors and/or pathways that regulate toxins or virulence factors production. In this review, we describe various *S. aureus* toxins and the major regulatory systems involved in the production of these toxins. We also address the potential of targeting *S. aureus* toxins and virulence-mediated pathways as anti-virulence strategies, in contrast to traditional antibiotics directed at pathogen viability.

## 2. Toxins—the Major *S. aureus* Virulence Factor

The versatility of *S. aureus* to survive host immune responses and cause a diverse range of diseases has been attributed to its ability to express a comprehensive repertoire of virulence determinants. The pathogenesis of *S. aureus* infections depends on the production of surface proteins that mediate bacterial adherence to host tissues, secretion of a series of extracellular toxins, and enzymes that destruct host cells and tissues, avoidance of, or incapacitating, the host immune defense, and growth and spread of bacteria in host cells [2]. Toxins are proteins secreted by *S. aureus* into the extracellular matrix during the post-exponential and early stationary phases. These proteins are usually involved in tissue penetration and enable the bacteria to invade its host. They are also cytolytic and help bacterial growth by acquiring essential nutrients such as iron from lysed-cells. Amongst the more common toxins secreted by *S. aureus* are hemolysin, leukotoxin, exfoliative toxin, enterotoxin, and toxic-shock syndrome toxin-1 (TSST-1). Aside from toxins, staphylococcal virulence factors also include enzymes and surface proteins. Secretion of enzymes, such as coagulase, proteases, and staphylokinase, helps in the bacteria’s evasion of host defenses, as well as host tissue invasion and penetration. Most of these enzymes function via degradation of host molecules or interfering with signaling cascades and metabolic pathways in the host [3,4]. In addition, *S. aureus* surface proteins (clumping factors, fibronectin proteins, protein A, collagen adhesin) also aid in bacterial adhesion, tissue invasion, and host defense evasion [5]. MSCRAMMS (microbial surface component recognizing adhesive matrix molecules) constitute the largest family of surface proteins and are integral for host extra-cellular matrix attachment and colonization [6,7]. For additional information of other virulence factors, readers are referred to previous reviews [5,8,9,10].

### 2.1. Hemolysins (Alpha, Beta, Gamma, and Delta)

Hemolysins are toxins that lyse red blood cells and their action is usually receptor-mediated. There are many classes of hemolysins, including α, β and γ-hemolysins. δ-hemolysin has been classified as a phenol-soluble modulin (PSM) that does not require a receptor for its hemolytic activity. α-hemolysin is the most well studied member of staphylococcal hemolysins. This small β-barrel pore-forming cytotoxin lyses red blood cells and leukocytes, but not neutrophils [11], via binding to its proteinaceous receptor ADAM10, a disintegrin and metalloproteinase [12]. In sepsis, synergistic action of α-toxin on myeloid cells and platelets has been shown to kill host animals and ADAM10 knockout models appear to be protected from the lethal effects of this toxin [13]. Upon binding of the toxin with its receptor, pore formation on cell membranes will cause Ca^2+^ influx and K^+^ efflux; this disruption in homeostasis, in turn, leads to necrotic cell death. β-hemolysin is non-pore-forming and has been characterized as a sphingomyelinase. The toxin hydrolyses sphingomyelin and also lyses monocytes; however, it only lyses erythrocytes at low temperatures and is not cytolytic to lymphocytes and granulocytes [14]. Even though its target cells are known, the toxin’s mode of action is still unclear. It has been postulated that as β-hemolysin acts mostly on sphingomyelin, the toxin most probably destabilizes the cells’ plasma membrane bi-lipid layer and causes irregularity in plasma membrane fluidity [15]. γ-hemolysin is hemolytic to rabbit erythrocytes and its membrane damaging activity is also apparent in leukocytes (neutrophils, monocytes, granulocytes, and macrophages) [15]. This group of hemolysins are bi-component and made up of polypeptides designated as S (slow, HlgA or HlgC) and F (fast, HlgB), where the S components are proposed to influence cell type susceptibilities to these toxins [16]. HlgB (F component) will bind to phosphatidylcholine on target cells [17] while its corresponding S component binds to host cell membranes, resulting in cell lysis [16]. PSMs (δ-hemolysin, PSMα1-4, PSMmec, PSMβ1-2) are multifunctional peptides in staphylococcal pathogenesis produced by many *S. aureus* strains; they are hemolytic to erythrocytes, various organelles, bacterial protoplasts, and spheroplasts [18]. The toxins are small and amphipathic with high affinity to lipids [15]. *S. aureus* PSMα has been shown to lyse neutrophils post-phagocytosis [19] and contributes to biofilm formation [20].

### 2.2. Leukotoxins

Leukotoxins lyse white blood cells and most likely require receptors for their mode of action although the receptors remained mostly uncharacterized until recently. This group of toxins is from the bi-component Luk toxin family and consists of Panton-Valentine leukocidin (PVL) (LukS and LukF proteins), LukDE, and LukAB (also known as LukGH), with PVL reported to be 100-fold more potent than the others. In animal models, LukS-PV and LukF-PV bind to TLR2 and TLR4 [21,22] whilst, in humans, the receptor for LukED is CCR5, whereas the receptors for PVL and LukAG/GH are C5aR, C5L2, and CD11b, respectively [23,24,25]. Lipids might act as co-receptors for LukS and LukF [15]. PVL has been reported to be associated with community acquired (CA)-MRSA infections, but this remains controversial [26].

### 2.3. Staphylococcal Exfoliative Toxins (ETs)

Staphylococcal exfoliative toxins (ETs) are serine proteases and the causative agent for staphylococcal scalded skin syndrome (SSSS), a disease that predominantly affects neonates and infants. Adults with renal impairment and immune deficiency are also at risk [27]. Infected individuals will experience blistering of the skin and loss of superficial skin layers, dehydration and secondary infections. Unlike other *S. aureus* toxins, the mode of action of ETs has been elucidated: ETs target the protein desmoglein 1 [28] and cleave this protein to destroy desmosomal cell attachments, causing epidermal detachment of skin epidermis [29]. Disruption of the skin epidermal layer further facilitates the progression of infection. ETs are also superantigens but are milder compared to other superantigens such as TSST-1 [30].

### 2.4. Staphylococcal Enterotoxins (SEs) and Toxic-Shock Syndrome Toxin-1 (TSST-1)

Staphylococcal enterotoxins (SEs) cause vomiting and diarrhea and the toxins are one of the most common causes of food-borne diseases. The toxins are secreted by entero-toxigenic *S. aureus* strains in food; they are heat-stable and are not degraded by cooking processes. There are more than 20 identified SEs to date: they are differentiated based on antigenic heterogeneity (SEA—SE*l*V) [31]. The SEs are superantigens which trigger T-cell activation and proliferation; their mode of action probably includes activation of cytokine release and cell death via apoptosis and potentially lethal toxic shock syndrome [32,33]. Nevertheless, even though the superantigenic activities of SEs have been well characterized, the mode of action(s) leading to emesis and diarrhea is still unclear. SEF has been renamed as TSST-1 (toxic-shock syndrome toxin-1) [34]. TSST-1 secretion leads to serious morbidity and mortality, for example, in tampon-associated TSST-1 cases [35]. Interestingly, the gene encoding this toxin is carried by only a limited number of strains. The lethality of TSST-1 does not seem to depend on T-cell proliferation but instead involves other types of host cell receptors. It has been reported that TSST-1 will stimulate the release of chemokines, such as IL-8 and MIP-3α, IL-2, and TNFα [36,37]. Activation of immune cells will enhance inflammation and cause mucosal cell barrier disruption, allowing further interaction of the toxin with T-cells and macrophages, leading towards toxic shock syndrome [38].

## 3. Regulation of Toxin Production in *S. aureus*

How *S. aureus* virulence gene expression is regulated is critical in defining the pathogenesis of staphylococcal diseases. Expression of the exoprotein genes is primarily orchestrated by a complex global regulatory system known as accessory gene regulator (Agr) [39] which responds to environmental signals such as bacterial cell density (quorum sensing), nutritional status, and energy availability [40]. Apart from the staphylococcal *agr* global regulator, several other regulatory systems have also been found to control the production of virulence determinants in *S. aureus*, including the Staphylococcal accessory regulator A (SarA) [41], *S. aureus* exoprotein expression (Sae) operon [42], and the Staphylococcal alternative sigma factor B (SigB), which act either coordinately or independently. The fine control of toxin production by *S. aureus* is a complicated process and has not yet been fully elucidated. The following section discusses the role of these regulators in directing the expression of virulence determinants.

### 3.1. The Two-Component Regulatory Systems—Agr and Sae

The *agr* is currently the most extensively studied global regulator of virulence determinant production in *S. aureus.* The production of vital *S. aureus* toxins such as α-, β- and γ-hemolysins, TSST-1, enterotoxin B, D, and C, exfoliatin A and B and PVL is positively regulated by the Agr system [43,44,45,46,47,48]. In addition to toxins, the secretion of various *S. aureus* enzymes such as serine protease, V8 protease, staphylokinase, glycerol ester hydrolase, nuclease, lipase, PI-phospholipase C and fibrinolysin was also shown to require a functional *agr* system for maximal expression [39,49,50,51]. Genes regulated by *agr* were validated by transcription profiling of an *agr* mutant [52]. The *agr*-dependent exoprotein secretion occurs at the end of the exponential phase and continues on into stationary phase [39].

**Figure 1:**
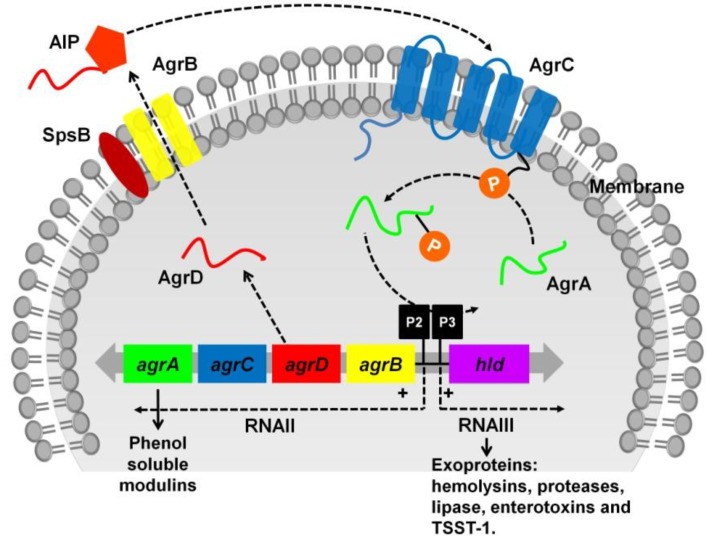
Schematic diagram of the *S. aureus agr* regulatory system. The *agr* operon consists of two transcriptional units RNAII and RNAIII, driven by the promoters P2 and P3, respectively. RNAII is an operon of four genes, *agr BDCA*, encoding AgrB responsible for processing and exporting AgrD, the AIP precursor. At threshold levels of AIP, AgrC will be autophosphorylated, leading to the phosphorylation of AgrA. AgrA activates RNAIII expression, thereby increasing the secretion of *S. aureus* toxins and enzymes.

The *agr* locus is part of the *S. aureus* core genome and as outlined in Figure 1, is composed of two adjacent but divergent transcriptional units directed by two promoters, P2 and P3 [53]. P2 controls the transcription of RNAII, an operon of four genes, *agrBDCA,* encoding the core machinery of the *agr* system while P3 drives the transcription of RNAIII, a regulatory RNA of the *agr* system. The transmembrane endopeptidase AgrB, together with SpsB, processes and cyclizes AgrD, the peptide precursor of the autoinducer peptide, AIP, into an octapeptide, and exports it via AgrB [54]. AgrC and AgrA are homologs of histidine protein kinase sensors and response regulators that form a classical bacterial two-component signal transduction system [55,56]. At the threshold concentration, AIP binds to the extracellular receptor on AgrC. AgrC will undergo autophosphorylation and subsequently phosphorylates AgrA into its active form which, in turn, triggers P2 and P3-driven transcription followed by translation of RNAIII [57] which leads to an up-regulation of toxins and enzymes such as hemolysins, proteases, lipase, enterotoxins, and TSST-1. Binding of AgrA to the promoters also enhances the transcription of *RNAII*, creating a positive feedback loop to the *agrBDCA* operon [53,56]. Additionally, AgrA can turn on transcription of the two promoters for expression of PSMs independent of RNAIII [58]. RNAIII controls the expression of virulence factors at both transcriptional and translational levels [56]. The coding region of the δ-hemolysin encoding gene *hld*, is embedded within the region encoding RNAIII [59] (Figure 1).

The sae locus encodes a second major two-component regulatory system which is involved in the control of *S. aureus* toxins and other exoproteins expression [42]. The two genes saeR and saeS encode a response regulator and a histidine protein kinase, respectively [60]. Microarray analysis comparing the transcriptomic profile of wild-type *S. aureus* and a saeR/S mutant revealed a subset of 80 genes and 133 genes which were up- and downregulated, respectively, in the saeR/S deletion mutant [61]. Among the virulence factor-encoding genes down-regulated in the saeR/S mutant were genes encoding α- and γ-hemolysins, PVL, staphylococcal exotoxin, staphylococcal enterotoxin, proteases, nuclease, coagulase, and fibrinogen-binding proteins [61,62,63]. The saeR/S system was deemed to be essential for full pathogenesis of *S. aureus* in multiple animal models of infection [61,62,64]. As a sae mutation did not affect the expression of agr, it is proposed that the saeR/S system modulates the production of virulence factors downstream of agr [65,66]. For additional discussion on the *S. aureus* virulence regulatory network, readers are referred to these comprehensive reviews [67,68].

### 3.2. The sarA and sigB

SarA is a global transcriptional regulator that has been studied in considerable detail since its discovery over two decades ago [41]. Through genome-wide transcriptional profiling, over 120 genes are known to be modulated by the *sarA* locus [52]. SarA-upregulated genes encode virulence factors such as β- and δ-hemolysins, staphylococcal exotoxin, and most of the cell-bound proteins including fibrinogen, fibronectin-binding proteins, and extracellular protein A; whilst genes downregulated by SarA encode for lipase, nuclease, serine protease, α-hemolysin, as well as aureolysin [41,52]. Expression of *sarA* is also positively correlated with *S. aureus* biofilm formation [69] as validated by reduced biofilm formation in *sarA* mutants, leading to an increase in bacterial susceptibility towards antimicrobial agents [70]. The expression of DNase and β-lactamase remains unaffected in Sar mutants [41].

The *sarA* locus contains a 372-bp ORF with three distinct promoters (P1, P3, and P2) driving three overlapping transcripts with different sizes coding for the SarA protein [71]. SarA can act directly by binding to the conserved sequence within the promoter region of target genes and trigger the transcription of exoproteins (toxins), as well as cell surface proteins (protein A and fibronectin-binding proteins) [72]. It can also function indirectly through a downstream effect on other regulators such as binding to the P2 promoter of the *agr* system [73], thereby activating the transcription of *RNAII* [74]. Inactivation of *sarA* attenuated *S. aureus* virulence in several experimental animal models [75,76,77].

The sigma factor in *S. aureus* interacts with RNA polymerase to form a holoenzyme that recognizes specific promoter sequences, leading to transcription initiation. Sigma factor B belongs to a chromosomal operon of four genes, namely *rsbU*, *rsbV*, *rsbW*, and *sigB* [78]. In unstressed conditions, SigB is bound to RsbW and, therefore, transcription is not initiated. Once the bacteria is stressed, RsbV will be dephosphorylated by RsbU prior to its interaction with RsbW; this releases SigB and activates transcription [79]. *SigB* activity is positively correlated with *sarA* expression and this trend is also observed in toxins regulated by the Sar regulator, including TSST-1 and β-hemolysin [80,81]. The inverse correlation between *sigB* and *agr* was also recently reported in regulation of TSST-1 expression [80], and this divergence in expression was also observed in staphylococcal enterotoxin B expression [82]. The role of SigB in *S. aureus* virulence has been demonstrated in a few infection models, including *S. aureus*-induced arthritis and sepsis models [83] and the rat chronic osteomyelitis model [84].

## 4. From Antibiotics to Anti-Virulence Therapies

The discovery of antibiotics is one of the greatest medical breakthroughs of the early twentieth century and today, treatment of bacterial infections still relies heavily on the use of antibiotics. Antibiotics disrupt the bacterial growth cycle by blocking the synthesis and assembly of key factors required for survival. Although antibiotics are highly effective, inappropriate, or overuse has led to bacterial antibiotic resistance. Uncontrolled use of antibiotics also destroys the beneficial protection provided by the gastrointestinal microflora population in the host, making the host susceptible to infection by commensal microorganisms [85]. To make matters worse, the discovery and development of new antibiotics is waning and the number of innovative drugs reaching the market has lagged far behind the growing demand.

The major events in the history of *S. aureus* antibiotic resistance are typified by the emergence and rampant rise of penicillin-resistant *S. aureus* (PRSA), the increased prevalence of MRSA including both hospital acquired- (HA-) and community acquired (CA)-MRSA, the recognition of vancomycin-intermediate *S. aureus* (VISA), followed by the emergence of vancomycin-resistant *S. aureus* (VRSA). Recently, the World Health Organization (WHO) initiated the very first World Antibiotic Awareness Week to create awareness of the global threat of antibiotic resistance, as well as to address the challenges concerning the misuse of antibiotics in clinical practice [86]. The emergence and spread of antibiotic resistance, exemplified by the emergence of new and variant pathogens, further highlights an urgent need for novel therapeutic drugs that do not exert selective pressure that leads to evolutionary adaptation of the bacteria. An alternative approach is to develop anti-virulence therapies that interfere with bacterial virulence determinants (e.g. toxins, enzymes, or surface proteins) and/or pathways that mediate virulence (e.g. two-component virulence regulatory systems, global transcriptional regulators, or quorum sensing system) that would less likely promote the development of antibiotic resistance. Extensive knowledge on how bacterial pathogens establish infection and increased understanding of genomic determinants of pathogen virulence have opened a path to tailor robust novel anti-virulence therapeutics.

Anti-virulence compounds offer an attractive option to conventional antibiotics and hold great promise as a new therapeutic paradigm. One of the pivotal characteristics of an anti-toxin or anti-virulence compound is that the compound does not affect bacterial viability or growth. Conceptually, anti-virulence therapies that do not target bacterial viability are likely to target nonessential genes and impose reduced selective pressure minimizing the probability of resistance development [87]. Since the compound does not target bacterial growth, this strategy ensures the preservation of the host endogenous microflora population. It is believed that by inhibiting bacterial virulence traits, the bacteria are less able to colonize the host and this may allow the host natural immunity to eradicate the attenuated pathogen.

A study by Mellbye and Schuster noted that anti-virulence drugs can potentially suppress bacterial resistance whereby in pathogens that rely on cell-to-cell communication (such as *S. aureus* and *Pseudomonas aeruginosa*), resistant strains will not outcompete susceptible strains, thereby allowing anti-virulence therapies that target this social communication network to be effective even when resistance arises [88]. Allen *et al.* [89] proposed that a combination of anti-virulence drugs that target different types of virulence factors together with a proper treatment environment would be a more effective solution.

### 4.1. Targeting S. aureus Toxins: A Direct Approach

Targeting toxins has been a successful approach in combating infections against a number of pathogens including *Bacillus anthracis, Bordetella pertussis*, and *Clostridia spp*. In this particular section, we discuss the potential therapeutic agents that inhibit *S. aureus* hemolysins, leukotoxins, and enterotoxins via various modes of action. A number of *S. aureus*-specific anti-toxins have shown promise in animal models of infection. However, to date, none have been successful in clinical trials, although ample pre-clinical evidence exists that anti-toxin neutralizing agents are highly effective in preventing or treating a wide range of diseases caused by *S. aureus*.

#### 4.1.1. Targeting *S. aureus* Hemolysins

The approval for the use of monoclonal antibodies (mAbs) against anthrax toxin by the FDA in 2009 led to studies exploring the potential of mAbs as a therapeutic option for *S. aureus* toxins. As described above, hemolysins are major *S. aureus* toxins expressed by most *S. aureus* strains. Hemolysin, in particular α-hemolysin (Hla), has received substantial attention as a target for anti-toxin neutralizing antibodies. Anti-α-hemolysin antibodies confer a high degree of protection against lethal staphylococcal pneumonia caused by diverse *S. aureus* clinical isolates in experimental animals [90,91] and significantly reduced abscess formation in a *S. aureus* dermonecrosis model [92,93]. Moreover, the therapeutic efficacy of antibodies appears to be additive or synergistic when applied in combination with clinically used antibiotics (vancomycin or linezolid) [91,93], signifying that the presence of these antibodies makes treatment by antibiotics more efficacious. A β-hemolysin neutralizing single-domain antibody that inhibits Hlb hemolytic activity *in vitro* has been isolated by antibody phage display [94]. Recently, a single antibody with high affinity and cross-reactivity towards α-hemolysin and 4 bi-component leukocidins was shown to prevent destruction of multiple human cells by both these toxins [95].

In addition to neutralizing antibodies, a number of molecules or compounds that block the hemolytic activity of *S. aureus* α-hemolysin have been discovered over the last few years. One example is β-cyclodextrin derivatives, small molecules that have been reported to inhibit α-hemolysis *in vitro* [96] and protect the host during a *S. aureus* infection [97,98]. The use of *in silico* tools and simulation program have facilitated the discovery of other α-hemolysin inhibitors [99,100,101,102]. The availability of the α-hemolysin crystal structure has enabled high throughput virtual screening of peptide or compound libraries for potent inhibitors [103,104]. For toxins with known host receptors, therapies can be designed to antagonize the receptor. ADAM10, the zinc-dependent metalloprotease receptor for Hla [12], is an attractive target for the design of an inhibitor to block toxin-receptor binding [105] to reduce lesions and the severity of recurrent skin and soft tissue infections [106]. Nevertheless, ADAM10 is a member of a large family and the role of ADAM10 has not been fully understood for other conditions, cautioning the use of an ADAM10 inhibitor.

#### 4.1.2. Targeting *S. aureus* Leukotoxins

Although its contribution in CA-MRSA pathogenesis is still controversial, PVL remains as one of the main targets for the development of anti-toxin therapies. The commercially available human intravenous polyclonal immunoglobulin preparations (IVIg) contain PVL-specific antibodies and exhibit anti-PVL leukotoxicity in a dose-dependent manner, probably through interfering with PVL-neutrophil binding [107]. Recently, in a 3D human lung tissue model, IVIg, abrogated recombinant PVL and α-toxin-mediated damage in epithelial cells [108]. Proper antibiotic treatment in combination with IVIg anti-toxin therapy markedly improved the condition of patients with severe necrotizing pneumonia caused by PVL-positive *S. aureus* strains [109], further supporting the use of IVIg in halting disease progression especially in highly lethal *S. aureus* infections associated with PVL production. Leventie *et al.* produced humanized heavy-chain-only mAbs against LukS-PV and LukF-PV, as well as a bispecific tetravalent anti-PVL antibody that bound both LukS-PV and LukF-PV subunits [110]. These antibodies inhibit PVL binding to polymorphonuclear leukocytes and prevent the formation of new pores *in vitro*; similarly, in a non-infectious rabbit model of endophthalmitis, a decrease in inflammatory response and tissue injury was observed, with the tetravalent anti-PVL antibody showing a more promising effect [110]. It is worth noting that the anti-LukS-PV antibody was also able to cross-react and neutralize the HlgC subunit in γ-hemolysin, owing to the high degree of similarity in protein sequence within leukotoxins [110]. This suggests that broad-spectrum anti-toxin therapies can be generated against more than one member of the leukotoxin family. A polyclonal antibody generated against an attenuated LukS-PV mutant [111] neutralizes various leukotoxins including PVL, HlgAB, HlgCB, and LukED [112]. While an epidemiological correlation between PVL and CA-MRSA has been reported in some studies [113,114], the presence of high levels of neutralizing antibodies did not confer resistance towards skin and soft tissue-infections caused by PVL-positive MRSA [115]. Thus, the effective utility of anti-toxin antibodies against PVL requires further evaluation. In a recent study, an antimicrobial peptide (AMP) isolated from human neutrophils was identified as an anti-PVL candidate that protects leukocytes from the cytotoxic effect of PVL [116].

#### 4.1.3. Targeting Staphylococcal Enterotoxins

Staphylococcal enterotoxin B (SEB) is one of the most-studied enterotoxins and its classification as a select agent by the United States Department of Health and Human Services makes it an attractive target for the development of anti-toxin neutralizing antibodies. SEB is relatively stable, highly resistant to denaturation and broadly classified as a superantigen [117] that can trigger increased secretion of pro-inflammatory cytokines, such as IFN-γ and TNF-α [118]. Most of the experimental therapies that target SEB are mAbs that directly bind to and neutralize SEB’s interaction with host cell receptors, leading to a reduction in pro-inflammatory cytokine production and protective immunity against the toxic and lethal effects of the toxin [119,120,121,122]. The human monoclonal antibody HuMAb-154 [119] exhibits high specificity and binding activity towards SEB. This antibody is also able to neutralize SEB-induced cytokines and prolong the survival of SEB-challenged mice, implying its potential as a prophylactic and therapeutic measure during a *S. aureus* infection [119]. A murine immunoglobulin IgG1 (mAb 20B1) also demonstrated potent neutralization of SEB [123]; it protected the host from lethal MRSA sepsis, as well as reduced the bacterial burden and abscess formation in superficial skin and deep-tissue infection models [122].

More recently, utilizing both NMR and crystallography techniques, the underlying mechanism and exact interaction of mAb 20B1 and SEB have been fully elucidated [121]. SEB superantigens bind to the variable region of the T-cell receptor β chain (Vβ). Buonpane *et al.* generated high-affinity soluble Vβ proteins as Vβ antagonists that compete with the Vβ region on the host T-cell receptor for binding to SEB, thus neutralizing SEB-mediated T-cell activation both *in vitro* and *in vivo* [124]. Subsequently, a broad spectrum neutralizing agent effective against both SEB and TSST-1 was engineered and proven to be active *in vitro* [125], paving the way for a single agent that is active against multiple toxins. In addition, targeting the toxin-induced host inflammatory response with known anti-inflammatory drugs is a quick approach to mitigate the toxic effects triggered by SEB via inhibition of T-cell proliferation and release of pro-inflammatory mediators [126]. Table 1 summarizes the potential anti-*S. aureus* toxin candidates discussed in this review and their respective modes of action.

### 4.2. Targeting Pathways that Govern Toxin Production: An Indirect Approach

The diverse and functionally redundant *S. aureus* virulence factors, including toxins and enzymes, are regulated by an intricate network of two-component regulatory systems and global transcriptional regulators. The coordinated and temporal expression of various virulence factors is important for the bacteria to adhere to host cells, establish infection and cause tissue damage in the host. As the pathogenesis of *S. aureus* infection cannot be attributed to a single virulence determinant, attenuation or neutralization of individual virulence factors may not confer meaningful protection to the host. In addition to the approach of direct neutralization of toxins described in the previous sections, targeting *S. aureus* toxins can also be done indirectly via disrupting the regulatory mechanisms that control virulence expression. As discussed in Section 3, the major virulence regulators in *S. aureus* are the two-component signal transduction systems (Agr and Sae) and SarA transcriptional regulator. *agr* and *sarA* knockout mutants were significantly attenuated in animal models [52,75], underscoring the importance of targeting these vital regulons to render *S. aureus* avirulent or less virulent.

In lieu of its role as a key virulence regulator in *S. aureus*, the Agr system is a robust target for the development of anti-virulence therapy. The two main proteins in the Agr system are the AgrA response regulator and the AgrC histidine kinase. In response to increasing cell density or quorum-sensing signals, AIPs will be produced to bind to and activate AgrC, directing the subsequent activation of AgrA (Figure 1). AgrA binds to P2 and P3 promoter regions, stimulating the transcription of RNAII and RNAIII which are responsible for downstream toxin gene expression. As RNAIII is the effector molecule that controls the expression of a large number of virulence factors, inhibiting RNAIII is a favorable approach to reduce the production of toxins and other virulence factors. RNAIII-inhibiting heptapeptide (RIP) interferes with quorum sensing, reduces *S. aureus* virulence and reverses the overall pathology in various animal infection models [127,128]. Due to its strong effect in attenuating *S. aureus* virulence, various RIP derivatives have been synthesized and examined. Two novel RIP derivatives (RIP-V and RIP-L) were recently found to significantly prolong the survival of mice and improve the pathological injuries in an MRSA sepsis model without affecting bacterial viability [129]. In the presence of RIPs, downregulation of RNAIII expression diminished the production of hemolysin. In combination with clinically used antibiotics, RIP accelerated wound healing and minimized the lethality rate in a *S. aureus*-induced sepsis mice model compared to treatment with antibiotics alone [130,131]

**Figure 2:**
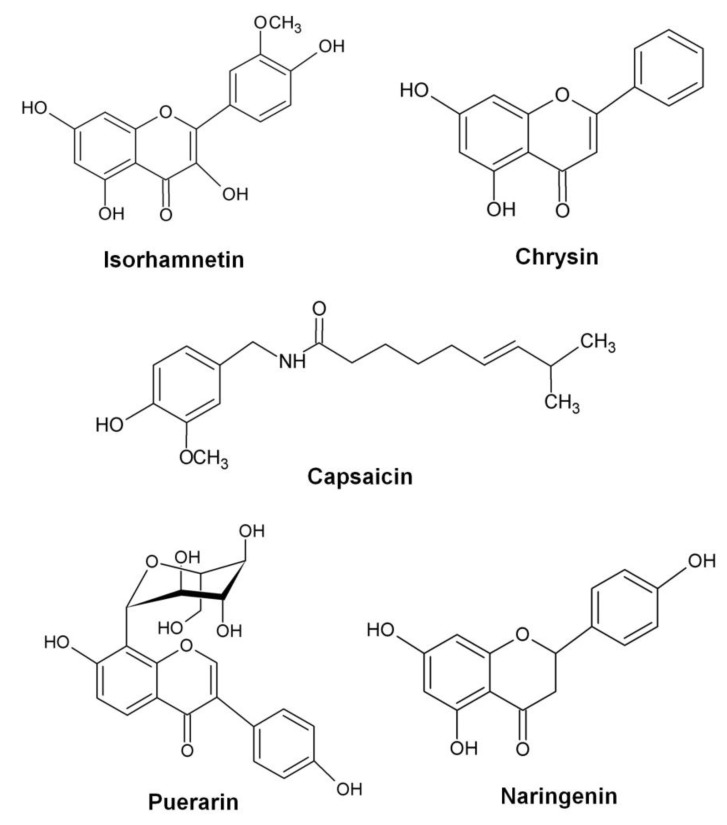
Chemical structures of natural compounds that exhibit anti-virulence properties (redrawn from [132,133,134,135,136]).

A number of bioactive components isolated from natural products, including isorhamnetin, chrysin, capsaicin, and puerarin (Figure 2), decreased *RNAIII* expression and subsequently *hla* expression which successfully protected the host from pneumonia caused by both MRSA and MSSA [132,133,134,135]. A significant reduction in *agrA* and *hla* transcript levels was detected in post-exponential *S. aureus* culture upon treatment with sub-inhibitory concentrations of a natural compound, naringenin (Figure 2). This compound also reduced the production of α-hemolysin and protected mice from *S. aureus*-provoked pneumonia [136]. With the exception of capsaicin, all other compounds are flavonoids that consist of a 2-phenyl-1,4-benzopyrone (flavone) backbone (Figure 2). Although the underlying molecular mechanism of these chemical entities has yet to be established, these compounds represent potential new scaffolds for the development of viable leads from natural products as anti-virulence agents in a *S. aureus* infection.

**Figure 3:**
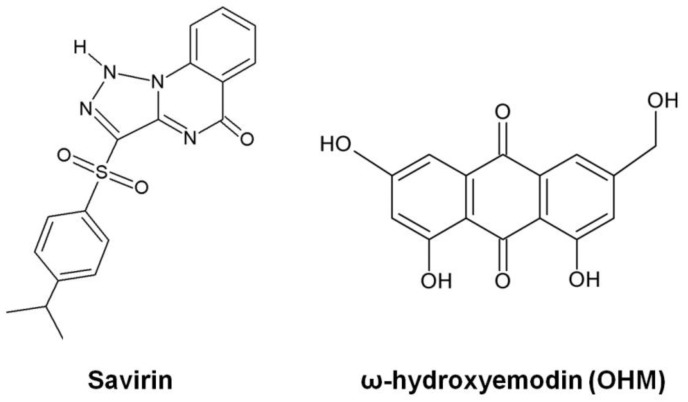
Chemical structures of savirin and OHM (redrawn from [138,139]).

High-throughput virtual screening of small molecule libraries for AgrA inhibitors identified a number of compounds that did not affect bacterial growth, but, were able to repress the expression of α-hemolysin, PSM, and to some extent RNAIII, by preventing specific binding of the AgrA response regulator to the P3 promoter [137]. In another high-throughput screen utilizing a transgenic *S. aureus* strain carrying *agr::P3* fused to a GFP reporter, Sully *et al.* identified a highly potent anti-virulence compound termed savirin (Figure 3) [138]. Savirin disrupted the Agr system by targeting the transcriptional function of AgrA, leading to downregulation of *agr*-regulated genes (including genes encoding hemolysin, PSM, PVL, lipase as well as protease) and production of secreted virulence factors. More importantly, savirin demonstrated high efficacy in two murine models of skin infection and repeated exposure to this compound had minimal impact on the development of drug resistance or tolerance [138]. The group extended the search for *agr*-quorum sensing inhibitors from their collection of natural bioactive compounds and successfully identified a second compound, ω-hydroxyemodin (OHM) (Figure 3), isolated from the fungus *Penicillium restrictum* [139]. This compound limited abscess and ulcer formation in a mouse model of skin and soft-tissue infection by binding to Agr and preventing AgrA from interacting with *agr* promoters, causing a reduction in secreted toxins and other virulence factors.

**Figure 4:**
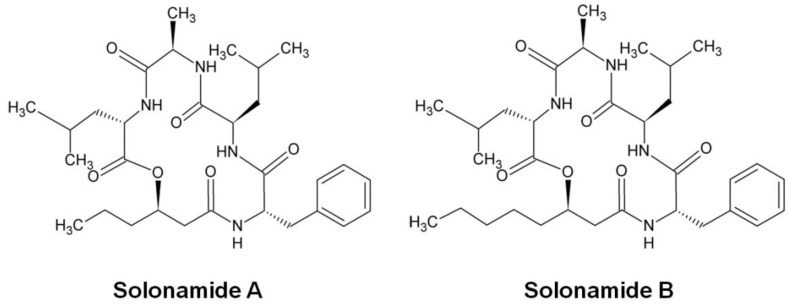
Chemical structure of solonamides isolated from *Photobacterium* sp. (redrawn from [140]).

In the search for new anti-virulence compounds that interfere with *agr* activity, Mansson *et al.* isolated two desipeptides, Solonamide A and Solonamide B (Figure 4), from a marine *Photobacterium* strain that increased the expression of *S. aureus* protein A and decreased the expression of *hla* and *RNAIII* [140]. The solonamides are structurally similar to AIPs and are thought to antagonize the *agr*-quorum sensing system in *S. aureus*. Further studies showed that Solonamide B reduced the transcription and activity of α-hemolysin by competition with AIPs for the binding to AgrC histidine kinase, thus compromising the activation of AgrC in various *S. aureus* strains, including the highly virulent CA-MRSA strain USA300 [141]. At the same time, this compound also dramatically suppressed the transcription and production of PSM, which is under the control of AgrA [141]. Other active ingredients isolated from marine microorganisms also influence the expression of *agr*-mediated *S. aureus* virulence genes, albeit to a lesser extent than solonamides [142,143].

A screen of over 12 flavonoids for anti-virulence activity against *S. aureus* demonstrated that flavone, the backbone compound of flavonoids, significantly suppressed the production of golden pigment staphyloxanthin and α-hemolysin [144]. Transcriptional analysis of flavone-treated *S. aureus* cells further validated the reduction in the expression of *hla* encoding α-hemolysin. Furthermore, flavone repressed the expression of *sae*, whilst elevating *agr* transcription but did not affect the transcript level of *sigB* and *sar,* suggesting that flavone exerts its anti-virulence effect via the regulation of *sae* and *agr* activity [144]. Proteomics and genomics studies of manuka honey-treated MRSA disclosed a downregulation of virulence-associated genes encoding α- and γ-hemolysin, enterotoxin C, lipase, and fibronectin-binding proteins, as well as a general reduction in the expression level of global virulence regulators, such as *agr*, *sae*, and *sarV* [145]. These findings offer the possibility of using medicinal compounds from natural sources to alter pathogenicity of antibiotic-resistant bacteria and future studies should focus on elucidating the anti-virulence mechanism of manuka honey in an animal model.

**Figure 5:**
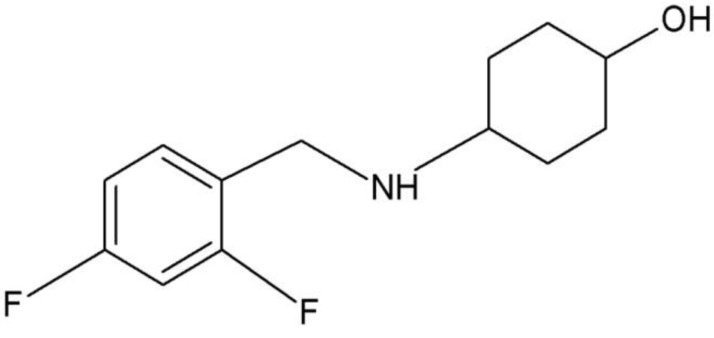
Chemical structure of SarABI (redrawn from [146]).

SarA is one of the most extensively studied *S. aureus* transcriptional regulators. Apart from *agr*- system inhibitors, targeting SarA-DNA interaction is a novel approach to combat *S. aureus* infections. Using a structure-assisted drug design approach, Arya *et al.* designed and synthesized a SarA inhibitor, SarABI (Figure 5) [146]. Interestingly, despite having no anti-bacterial activity, this compound demonstrated anti-biofilm effect, reduced hemolytic activity, and attenuated bacterial adherence to host cells. Furthermore, SarABI also drastically decreased the expression of *RNAIII*, *hld* and the fibronectin encoding gene *fnbA* and prevented vascular graft infection by *S. aureus in vivo* [146]. The selective binding of SarABI to the DNA binding domain of SarA was confirmed by docking analysis [146]. Collectively, this study highlights the potential of targeting SarA as a contemporary approach for development of a novel anti-virulence agent against *S. aureus*.

## 5. *Caenorhabditis elegans* as a Model for the Discovery of Novel Anti-Virulence Molecules

Targeting *S. aureus* toxins or virulence factors appears to be a key approach for future drug design. However, a major constraint is the difficulty in probing virulence factors and infection processes within the context of a human infection. In recent years, the establishment and description of a wide range of bacterial and fungal pathogen infections in *C. elegans* have prompted laboratories to adopt worms as an *in vivo* model for the discovery of anti-infectives. Infection of *C. elegans* by both MSSA and MRSA has been used as a robust platform to decipher the mechanisms of host-pathogen interaction [147,148]. The virulence determinants of *S. aureus* thought to be important in mammalian pathogenesis are also required for pathogenicity in *C. elegans* [147]. *S. aureus* mutants for crucial mammalian virulence factors, such as *agr* and *sarA*, are attenuated in *C. elegans* killing, indicating that these regulators and their downstream targets, for example hemolysin and V8 protease, are required for full pathogenesis in the *C. elegans* model. This also suggests that key aspects of *S. aureus* pathogenesis are highly conserved, irrespective of the host. To identify new therapeutic molecules towards *S. aureus* with a mode of action that is distinct from conventional antibiotics, our laboratory developed an *in vivo C. elegans—S. aureus* liquid-based screen to identify compounds that do not kill the pathogen but, in turn, abrogate *S. aureus* virulence in the context of a whole organism [149]. Screening of a collection of natural extracts and synthetic compounds identified a subset of viable leads that promoted the extended survival of both MSSA- and MRSA-infected nematodes without conferring anti-bacterial effects on the pathogen.

The advantages of using worms in a rapid screen are its simplicity and genetic tractability as an intact host model, inexpensive to cultivate as compared to other host models, easy manipulation, and short life cycle. The nematodes are easy to manipulate because they are small enough to fit into standard 96- or 384-well microtiter plates, making them easily adaptable for high-throughput screening of compound libraries. Furthermore, the *C. elegans* whole-animal approach provides early and direct assessment of drug efficacy *in vivo*, thereby filtering out compounds that are toxic to the host or with poor pharmacokinetic properties, at the initial stage of the screen [150]. Unlike the use of murine or rabbit models for drug testing, using the *C. elegans* model for drug discovery does not raise any ethical concerns. With an increasing understanding of host-pathogen interactions and bacterial pathogenesis, the *C. elegans* model presents an advantage in being able to simultaneously detect compounds that target bacterial virulence, as well as infection processes that are only manifested when the complex host-pathogen relationship is intact. As the underlying innate immunity signaling pathways recognized in *C. elegans* are evolutionarily conserved, it is conceivable that findings from the nematode model could be extended to higher organisms, including humans [151].

## 6. Concluding Remarks

Although there is currently no anti-virulence agent approved for clinical use against bacterial infections, an emerging body of evidence has shed light on the advantages and usefulness of these therapeutic agents for fighting infections; in particular, infections by antibiotic resistant strains. Direct or indirect approaches to target *S. aureus* toxins should lead to the development of new adjuvants or adjunct therapies to complement the use of antibiotics. Although efforts to exploit novel anti-virulence candidates have been met with varying degrees of success, there is a lack of pharmacology and toxicology data for the promising leads. Future work should focus on selected highly-effective candidates for detailed mechanistic analysis. The possible side effects of these anti-toxin or anti-virulence candidates should also be evaluated. One potential shortfall of anti-virulence agents is the potential narrow spectrum efficacy as these drug candidates target specific virulence-mediated pathways in selected pathogens, which may restrict its application in the clinical setting. On the other hand, this could be advantageous in terms of limiting the elimination of the beneficial microflora population (for a related review, see reference [152]). Hence, the application of anti-virulence therapy in humans, and its ability to impede the problem of drug resistance without imposing strong selective pressure upon the bacterial population, requires much more extensive evaluation.

## Figures and Tables

**Table 1:** Therapeutic agents against *S. aureus* toxins and their proposed mechanism of action.

Toxin Target(s)	Type	Name	Mode of Action	Phase of Development	References
α-hemolysin	Monoclonal antibody	MAbs 7B8 and 1A9	Antagonizes toxin activity by inhibiting the formation of fully assembled α-hemolysin oligomer.	Testing in animal model (mice pneumonia model)	[90]
α-hemolysin	Monoclonal antibody	MAb 2A3.1 (and affinity-optimized 2A3 variant—LC10)	Neutralizes toxin and prevents toxin-mediated cell lysis via a blockade of α-toxin heptamer formation on erythrocyte membranes.	Testing in animal models (*S. aureus* dermonecrosis murine model and mouse model of *S. aureus* pneumonia)	[91,92]
α-hemolysin	Monoclonal antibody	MAb LTM14	Prevents binding of toxin to the plasma membrane of susceptible host cells.	Testing in animal models (mice pneumonia, skin and bacteremia models)	[93]
α-hemolysin	Chemical compound	β-cyclodextrin derivatives	Blocks the transmembrane pores formed by the toxin and terminates ion conductance through the pores.	Testing in animal models (*S. aureus* pneumonia mice model)	[96,98]
α-hemolysin	Natural compound	Oroxylin A, Oroxin A and Oroxin B	Binds to the “stem” region of α-hemolysin and restricts the conformational transition of toxin from monomer to oligomer.	*In vitro* assays	[99,100]
α-hemolysin	Chemical compound	Isatin-Schiff copper (II) complexes	Prevents the formation of ion channels by obstructing the constriction region of the α-hemolysin channel.	*In vitro* assays	[101]
α-hemolysin	Natural compound	Morin hydrate	Inhibits self-assembly of the heptameric transmembrane pore of α-hemolysin.	Testing in animals (mice pneumonia model)	[102]
α-hemolysin	Chemical compound	ADAM10 inhibitor (GI254023X)	Inhibits the binding of α-hemolysin to its receptor (ADAM10).	Testing in animals (mice model of recurrent skin and soft-tissue infection)	[106]
β-hemolysin	Single-domain antibody	dAb/SAE Cl-7-5	Neutralizes *S. aureus* Hlb activity.	*In vitro* assays	[94]
α-hemolysin and bi-component leukocidins	Monoclonal antibody	MAb Hla-F#5	Cross-neutralizes α-hemolysin and leukocidins by recognizing the conserved conformational epitope.	Testing in animal models (murine models of *S. aureus* pneumonia and bacteremia/sepsis)	[95]
PVL and α-hemolysin	Polyclonal antibody	Human intravenous polyclonal immunoglobuin (IVIg)—Tegeline	Inhibits the lytic effect of PVL on polymorphonuclear cells and neutralizes α-hemolysin.	*In vitro* assays for PVL; *in vivo* peritonitis murine model for hemolysin	[107,108]
PVL and γ-hemolysin	Humanized heavy chain-only antibody	Bivalent and tetravalent anti-PVL mAbs	Blocks binding of PVL to target cells and inhibits pore formation on target cells by γ-hemolysin.	Testing in animal models (rabbit model of toxin-induced endophthalmitis)	[110]
PVL and other leukotoxins	Polyclonal antibody	Anti-LukS-mut9	Cross-neutralizes the lytic activity of various leukotoxins on polymorphonuclear cells.	Testing in animal models (toxin-challenged mouse model)	[112]
PVL	Antimicrobial peptide	α-defensin HNP3	Binds to both LukS-PV and LukF-PV and reduces PVL-induced necrosis in human neutrophils by interfering with pore formation.	*In vitro* assays	[116]
SEB	Monoclonal antibody	HuMAb-154	Binds to SEB, neutralizes the toxin and inhibits SEB-induced production of proinflammatory cytokines.	Testing in animal models (mice model challenged by SEB)	[119]
SEB	Monoclonal antibody	MAb 20B1	Binds and neutralizes SEB.	Testing in animal models (mice sepsis, superficial skin and deep-tissue infection models)	[121,122]
SEB	Protein	Soluble Vβ protein	As a receptor antagonist that offers high-affinity binding to SEB superantigens and neutralizes the toxicity of SEB.	Testing in animal model (rabbit model of SEB-induced disease)	[124]
SEB and TSST-1	Protein	Broad spectrum Vβ protein	Binds to superantigens and neutralizes both SEB and TSST-1 activities.	*In vitro* assays	[125]
SEB	FDA-approved drug	Sulfasalazine	Reverses SEB-stimulated toxic effect by inhibiting the production of proinflammatory cytokines, T-cell proliferation and NFκB activation.	*In vitro* assays	[126]
